# IL-23 Inhibits Trophoblast Proliferation, Migration, and EMT via Activating p38 MAPK Signaling Pathway to Promote Recurrent Spontaneous Abortion

**DOI:** 10.4014/jmb.2112.12056

**Published:** 2022-05-17

**Authors:** Shan He, Yan Ning, Fei Ma, Dayan Liu, Shaoyan Jiang, Shaojie Deng

**Affiliations:** 1 Department of Pharmacy, Affiliated Shenzhen Maternity and Child Healthcare Hospital, Southern Medical University, Shenzhen, Guangdong 518028, P.R. China; 2 Department of Traditional Chinese Medicine, Affiliated Shenzhen Maternity and Child Healthcare Hospital, Southern Medical University, Shenzhen, Guangdong 518028, P.R. China; 3 Department of Genesiology, Affiliated Shenzhen Maternity and Child Healthcare Hospital, Southern Medical University, Shenzhen, Guangdong 518028, P.R. China

**Keywords:** Recurrent spontaneous abortion, IL-23, p38 MAPK signaling pathway

## Abstract

As a vital problem in reproductive health, recurrent spontaneous abortion (RSA) affects about 1% of women. We performed this study with an aim to explore the molecular mechanism of interleukin-23 (IL-23) and find optimal or effective methods to improve RSA. First, ELISA was applied to evaluate the expressions of IL-23 and its receptor in HTR-8/SVneo cells after IL-23 treatment. CCK-8, TUNEL, wound healing and transwell assays were employed to assess the proliferation, apoptosis, migration and invasion of HTR-8/SVneo cells, respectively. Additionally, the expressions of apoptosis-, migration-, epithelial-mesenchymal transition- (EMT-) and p38 MAPK signaling pathway-related proteins were measured by western blotting. To further investigate the relationship between IL-23 and p38 MAPK signaling pathway, HTR-8/SVneo cells were treated for 1 h with p38 MAPK inhibitor SB239063, followed by a series of cellular experiments on proliferation, apoptosis, migration and invasion, as aforementioned. The results showed that IL-23 and its receptors were greatly elevated in IL-23-treated HTR-8/SVneo cells. Additionally, IL-23 demonstrated suppressive effects on the proliferation, apoptosis, migration, invasion and EMT of IL-23-treated HTR-8/SVneo cells. More importantly, the molecular mechanism of IL-23 was revealed in this study; that is to say, IL-23 inhibited the proliferation, apoptosis, migration, invasion and EMT of IL-23-treated HTR-8/SVneo cells via activating p38 MAPK signaling pathway. In conclusion, IL-23 inhibits trophoblast proliferation, migration, and EMT via activating p38 MAPK signaling pathway, suggesting that IL-23 might be a novel target for the improvement of RSA.

## Introduction

Recurrent spontaneous abortion (RSA), defined as the loss of ≥2 consecutive pregnancies before the 24th gestational week, affects 1%-2% women during their reproductive age [1]. As a common condition in reproductive medicine, RSA is reported in 1-2% of fertile couples seeking pregnancy and inflicts a significant physical, emotional, and financial burden on many families [2]. Many factors are thought to contribute to RSA, such as parental chromosomal abnormalities, maternal thrombophilia, immune dysfunction, and various endocrine disorders [3]. In addition, the age of the pregnant mother has also been reported to be a strong independent risk factor for miscarriage, and in particular, the risk of fetal miscarriage greatly increases after 35 years of age [4]. However, in many cases, the causes of RSA could not be easily identified, while the evidence-based diagnostic and treatment strategies are in short supply and the prospects of treatment are not as promising as they could be [5].

Interleukin 23 (IL-23), which is composed of a p40 subunit and a specific p19 subunit, belongs to the IL-12 cytokine family and is secreted by dendritic cells and macrophages [6, 7]. Accumulated reports have confirmed that IL-23 participates in the development of some autoimmune inflammatory diseases, including psoriasis, arthritis, and inflammatory bowel disease, thus serving as a critical therapeutic target for the improvement of inflammatory diseases [8-10]. In addition, another study reported that IL-23 was upregulated in patients with RSA [11]. Moreover, both IL-17 and IL-23 were verified to inhibit Langerhans cell (LC) migration in a psoriasis mouse model [12]. We therefore speculated that IL-23 could inhibit the proliferation and migration of trophoblast cells.

The p38 mitogen-activated protein kinase (p38 MAPK), an evolutionarily conserved class of serine/threonine mitogen-activated protein kinases, was discovered in the mid-1990s [13, 14]. Being focal points for various extracellular stimuli, MAPKs function as a regulator in different cellular processes [15]. Moreover, once the p38 MAPK cascade is activated by pro-inflammatory and stressful stimuli, cellular responses such as inflammation, cell proliferation, differentiation, apoptosis and invasion can occur [16, 17]. Sudarshan Seshadri *et al*. showed that the identification of MAPK signaling in IL-23-mediated production of IL-22 might be a novel therapeutic approach, suggesting that IL-23 could activate the MAPK signaling pathway [18].

In this study, we sought to explore not only the role of IL-23 in RSA, but also its detailed molecular mechanism. In addition, HTR-8/SVneo (RRID: CVCL_7162), an immortalized human first-trimester trophoblast cell line, is useful in the study of trophoblasts and placental biology. Furthermore, in the extant studies on RSA, the HTR-8/SVneo cell line has been treated mostly as the subject of study [19-21]. Therefore, in our study, we employed HTR-8/SVneo cells in an attempt to find a potential therapeutic target for the improvement of RSA and provide new insights in the investigation of RSA.

## Material and Methods

### Cell Culture and Treatment

HTR-8/SVneo cells were provided by the Chinese Academy of Sciences Cell Bank (China) and incubated in Dulbecco’s modified Eagle medium (DMEM; Gibco, USA) containing 10% fetal bovine serum (FBS; Gibco, USA), 100 U/ml penicillin and 100 μg/ml streptomycin (Invitrogen, USA) in a humid atmosphere at 37°C with 5% CO_2_. R&D Systems (USA) supplied the recombinant human IL-23. Subsequently, HTR-8/SVneo cells were treated with IL-23 at different concentrations (50 ng/ml and 100 ng/ml [12, 18]). To explore the relationship between IL-23 and p38 MAPK signaling pathway, SB239063, a p38 MAPK-specific inhibitor, was also used for cell administration following the indicated treatment.

### Cell Counting Kit-8 (CCK-8)

After being seeding into 96-well plates for 24, 48, 72, and 96 h, HTR-8/SVneo cells were incubated with 10 μl of CCK-8 reagent for another 3 h at 37°C. Then, under condition of λ = 450 nm, the absorbance was evaluated with a microplate reader (Bio Rad, USA).

### Terminal Deoxynucleotidyl Transferase-Mediated Nick End Labeling (TUNEL)

With the application of the TUNEL assay, the apoptosis of HTR-8/SVneo cells was strictly assessed in line with the manufacturer’s protocol. After fixing with 4% paraformaldehyde for 30 min, the cells were permeabilized with 0.5% Triton X-100 and labeled with TUNEL for 1 h at 37°C. Following this, DAPI staining solution (Beyotime, China) was utilized to counterstain the sections for 5 min in the dark. Finally, the positive apoptotic cells were photographed by using fluorescence microscopy (Nikon , Japan).

### Western Blotting

Total proteins in HTR-8/SVneo cells after the indicated treatment were isolated with RIPA buffer. The protein concentration was detected by using a bicinchoninic acid (BCA) protein assay kit (Invitrogen, USA). After being subjected to a 12% gel sodium dodecyl sulfate polyacrylamide gel electrophoresis (SDS-PAGE), the proteins were transferred onto polyvinylidene fluoride (PVDF) membranes. The membranes were then impeded with 5% skim milk for 2 h followed by incubation with primary antibodies against Bcl-2 (ab32124; 1:1000; Abcam, China), Bax (ab32503; 1:1000; Abcam), cleaved-caspase3 (ab32042; 1:500; Abcam), MMP2 (ab92536; 1:1000; Abcam), MMP9 (ab76003; 1:1000; Abcam), E-cadherin (ab40772; 1:10000; Abcam), N-cadherin (ab76011; 1:5000; Abcam), p-p38 (mAb #4511; 1:000; Cell Signaling Technology, USA), p-ERK1/2 (mAb #4370; 1:2000; Cell Signaling Technology), p-JNK (mAb #4668; 1:1000; Cell Signaling Technology), p38 (mAb #8690; 1:1000; Cell Signaling Technology), ERK1/2 (mAb #8544; 1:1000; Cell Signaling Technology), JNK (ab76125; 1:1000; Abcam) and GAPDH (ab8245; 1:500; Abcam) at 4˚C overnight. Thereafter, the membranes were washed with tris-buffered saline and Tween (TBST) for three times, followed by incubation of membranes with HRP-labeled secondary antibody. Finally, the protein bands were captured by improved chemiluminescence (ECL, USA).

### Wound Healing

HTR-8/SVneo cells were inoculated into 6-well plates and cultured until the confluence reached 90-100%. In the cell monolayer, a linear scratch was made by a pipette tip. Then, phosphate-buffered saline (PBS) was used to wash the cells for three times to remove cell debris. After cell incubation at 37°C with 5% CO_2_, the width of the scratch was recorded and captured at 0 and 24 h. Finally, the relative migration rate of the HTR-8/SVneo cells was detected by Image-J software.

### Transwell

The relative invasive rate of the HTR-8/SVneo cells was assessed with the application of the transwell invasion assay. The upper chamber of the transwell was pre-coated with Matrigel (BD Biosciences, USA) and used for cell inoculation and culture. At the same time, medium containing 10% FBS was added to the lower chamber of the transwell 24 h later, and then the invading cells were fixed and stained with 4% paraformaldehyde and 0.1% crystal violet, respectively. Finally, images of the invaded cells were photographed under a microscope.

### Statistical Analysis

All data collected from our experiments were indicated as mean ± SD and analyzed with the help of GraphPad Prism 8.0 software (GraphPad software, Inc.). One-way analysis of variance (ANOVA) and Tukey’s test were used for comparisons among different groups. A *p*-value of <0.05 indicated statistical significance.

## Results

### IL-23 Inhibited the Proliferation of HTR-8/SVneo Cells

**Fig. 1:**
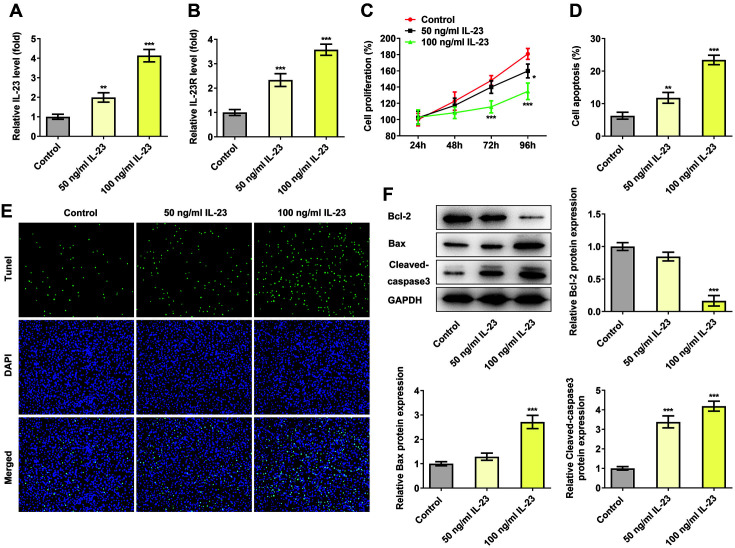
IL-23 inhibited the proliferation of HTR-8/SVneo cells. The expressions of IL-23 (**A**) and IL-23R (**B**) in IL-23- treated HTR-8/SVneo cells were detected using ELISA. (**C**) The cell proliferation of IL-23-treated HTR-8/SVneo cells was detected using CCK-8. (**D-E**) The apoptosis of IL-23-treated HTR-8/SVneo cells was detected using TUNEL. (**F**) The expressions of apoptosis-related proteins in IL-23-treated HTR-8/SVneo cells were detected using western blotting. *p < 0.5, **p < 0.01 and ***p < 0.001 vs. control.

ELISA assay was applied to detect the expressions of IL-23 and its receptors in IL-23-treated HTR-8/SVneo cells. As shown in Figs. 1A and 1B, the expression of IL-23 and its receptor was greatly elevated in IL-23-treated HTR-8/SVneo cells in comparison with the control. Notably, IL-23 with a concentration of 100 ng/ml contributed to higher expressions of IL-23 as well as its receptor in our experiments. The proliferation of HTR-8/SVneo cells was inhibited after IL-23 treatment. Significant proliferation inhibition was observed in 100 ng/ml IL-23-treated cells for 72 h and 50 ng/ml IL-23-treated cells for 96 h compared to the control group (Fig. 1C). Generally speaking, IL-23 exerted inhibitory effects on cell proliferation in a concentration-dependent manner. As shown in Figs. 1D and 1E, the apoptosis was greatly increased in HTR-8/SVneo cells after IL-23 treatment in contrast with the control, indicating the promotive effects of IL-23 on cell apoptosis. Moreover, compared with the control, IL-23 treatment downregulated Bcl-2 expression but upregulated the expressions of Bax and cleaved caspase-3 (Fig. 1F).

### IL-23 Inhibited the Migration, Invasion and Epithelial-Mesenchymal Transition Process of HTR-8/SVneo Cells

**Fig. 2:**
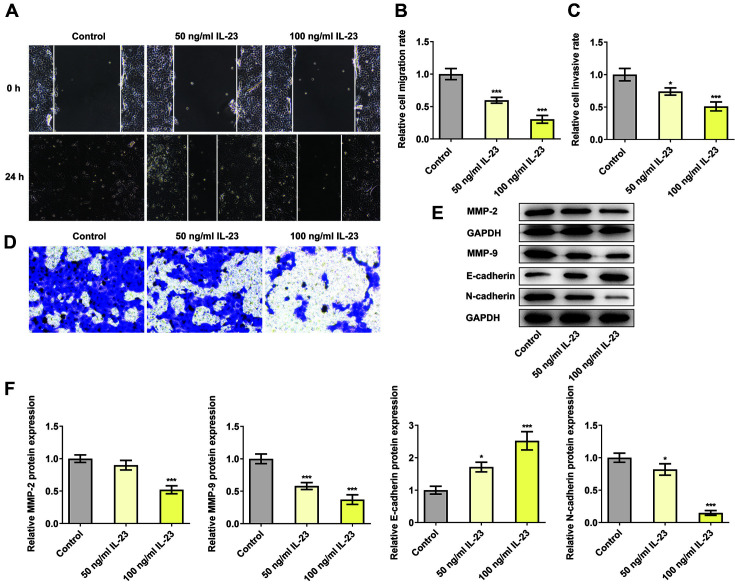
IL-23 inhibited the migration, invasion and epithelial-mesenchymal transition process of HTR-8/ SVneo cells. The migration (**A, B**) and invasion (**C, D**) of IL-23-treated HTR-8/SVneo cells were detected using wound healing and transwell assays, respectively. (**E, F**) The expressions of migration- and EMT-related proteins were measured using western blotting. *p < 0.5 and ***p < 0.001 vs. control.

By using wound healing and transwell assays, the migration and invasion of IL-23-treated HTR-8/SVneo cells were evaluated, respectively. According to the data from Figs. 2A-2D, the relative rates of migration and invasion were greatly decreased after IL-23 treatment in comparison with that in the control group, suggesting that IL-23 helped to suppress the migration and invasion of HTR-8/SVneo cells. Additionally, the expressions of migration-and EMT-related proteins including MMP2, MMP9, E-cadherin and N-cadherin, were measured by western blot. Clearly, IL-23 treatment upregulated E-cadherin expression but downregulated the expressions of MMP2, MMP9 and N-cadherin compared with that in control group (Figs. 2E and 2F). The previously mentioned results indicated the suppressive effects of IL-23 on cell migration and invasion as well as on the EMT process.

### IL-23 Activated p38 MAPK Signaling Pathway

**Fig. 3:**
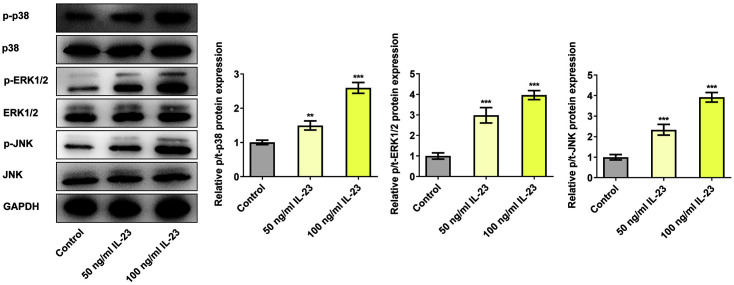
IL-23 activated p38 MAPK signaling pathway. The expressions of p38 MAPK signaling pathway-related proteins were detected using western blotting. **p < 0.01 and ***p < 0.001 vs. control.

With the aim of figuring out the relationship between IL-23 and p38 MAPK signaling pathway, the expressions of p38 MAPK signaling pathway-related proteins were measured by western blot. Obviously, the expressions of p38, ERK1/2 and JNK stayed unchanged after IL-23 treatment (Fig. 3). Nevertheless, IL-23 significantly upregulated the expressions of p-p38, p-ERK1/2 and p-JNK, which revealed that IL-23 could activate the p38 MAPK signaling pathway.

### IL-23 Inhibited the Proliferation of HTR-8/SVneo Cells via Activating p38 MAPK Signaling Pathway

**Fig. 4:**
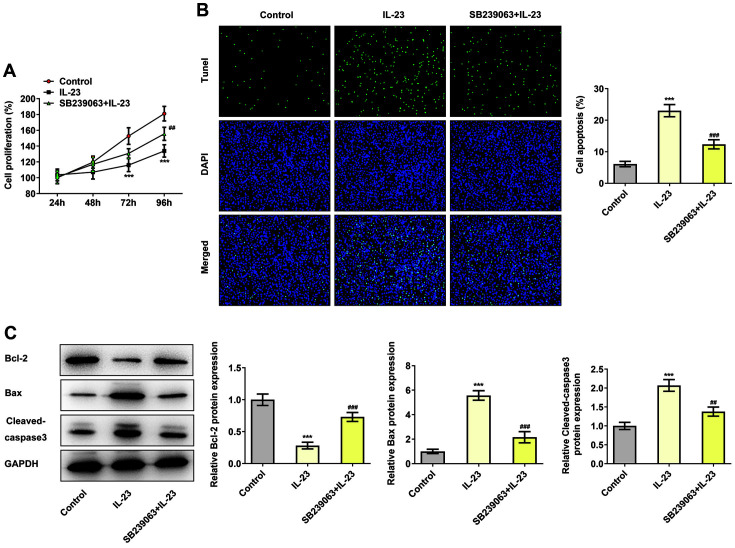
IL-23 inhibited the proliferation of HTR-8/SVneo cells via activating p38 MAPK signaling pathway. (**A**) The cell proliferation of IL-23-treated HTR-8/SVneo cells with SB239063 administration was detected using CCK-8. (**B**) The apoptosis of IL-23-treated HTR-8/SVneo cells with SB239063 administration was detected using TUNEL. (**C**) The expressions of apoptosis-related proteins in IL-23-treated HTR-8/SVneo cells with SB239063 administration were detected using western blotting. ***p < 0.001 vs. control, ^##^*p* < 0.01 and ^###^*p* < 0.001 vs. IL-23.

To explore the role of p38 MAPK signaling pathway in RSA, SB239063, a p38 MAPK-specific inhibitor, was applied to treat HTR-8/SVneo cells for 1 h at a concentration of 20 μM [22]. After that, a series of cellular experiments were conducted on cell proliferation and apoptosis as well as apoptosis-related proteins with the use of CCK-8, TUNEL and western blotting. As depicted in Fig. 4A, IL-23 treatment decreased the cell proliferation at 48, 72, and 96 h compared with control while SB239063 partially enhanced that decreased proliferation. In addition, the increased apoptosis in HTR-8/SVneo cells caused by IL-23 treatment was then suppressed by SB239063 administration (Fig. 4B). Moreover, SB239063 upregulated Bcl-2 expression but downregulated the expressions of Bax and cleaved caspase-3 in IL-23-administrated HTR-8/SVneo cells in contrast with IL-23 group (Fig. 4C). The above findings vividly suggested that SB239063 could reverse the promotive effects of IL-23 on cell apoptosis.

### IL-23 Inhibited the Migration, Invasion and Epithelial-Mesenchymal Transition Process of HTR-8/SVneo Cells via p38 MAPK Signaling Pathway

**Fig. 5:**
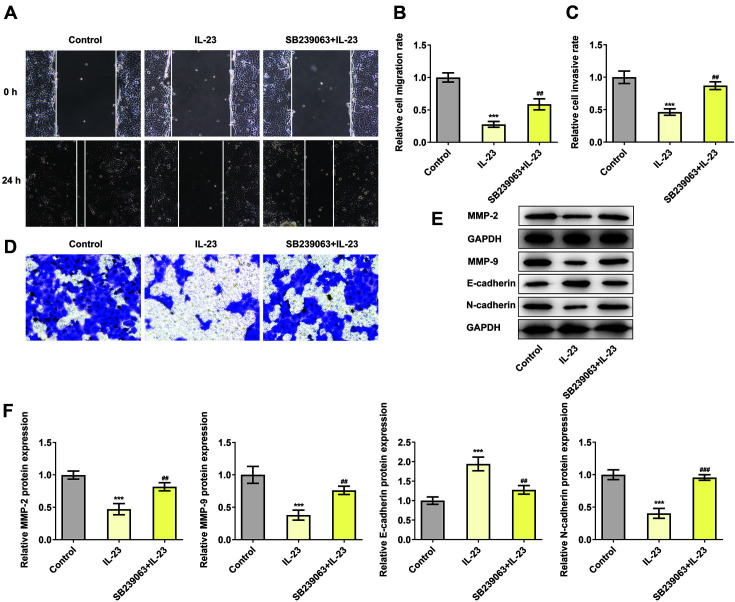
IL-23 inhibited the migration, invasion and epithelial-mesenchymal transition process of HTR-8/ SVneo cells via p38 MAPK signaling pathway. The migration (**A, B**) and invasion (**C, D**) of IL-23-treated HTR-8/SVneo cells with SB239063 administration were detected using wound healing and transwell assays, respectively. (**E, F**) The expressions of migration- and EMT-related proteins were measured using western blotting. ***p < 0.001 vs. control, ^##^*p* < 0.01 and ^###^*p* < 0.001 vs. IL-23.

The role of SB239063 in the migration, invasion and EMT development of IL-23-treated HTR-8/SVneo cells was also investigated in our study. Results obtained from wound healing and transwell assays implied that the decreased rates of relative migration and invasion induced by IL-23 administration were subsequently increased after SB239063 treatment (Figs. 5A-5D). Furthermore, IL-23 enhanced E-cadherin expression but reduced the expressions of N-cadherin, MMP2 and MMP9 compared with the control while SB239063 reversed these trends, evidenced by the downregulated E-cadherin expression as well as the upregulated expressions of N-cadherin, MMP2 and MMPs (Figs. 5E and 5F). The abovementioned results indicated that IL-23 exhibited suppressive effects on the migration, invasion and epithelial-mesenchymal transition advancement of HTR-8/SVneo cells through the activation of p38 MAPK signaling pathway.

## Discussion

Decidual macrophages, which can be found throughout all phases of pregnancy, were polarized as M1 subtype and released tumor necrosis factor-α (TNF-α), interleukin-6 (IL-6) and interleukin–1 beta (IL-1β) to promote RSA [23]. Previous studies have evidenced that pro-inflammatory cytokine IL-23 that secreted M1 macrophages were greatly elevated in patients suffering from RSA [11]. As far as we are concerned, this study was the first to explore the relationship of IL-23 and p38 MAPK signaling pathway in RSA. Here, we found that the expressions of IL-23 and its receptor were significantly upregulated in IL-23-treated HTR-8/SVneo cells. More importantly, our study revealed for the first time that IL-23 exerted suppressive effects on the proliferation, migration and EMT of trophoblast cells via activation of p38 MAPK signaling pathway, thus promoting the occurrence of RSA.

Successful embryo implantation depends on embryo incubation, trophectoderm development, proper maternal-fetal crosstalk and immune regulation [24]. Trophoblast cells are the most important cells in early pregnancy, and their proliferation, migration and invasion are essential for the establishment and maintenance of pregnancy [20]. It was reported that placental development mainly depends on the differentiation, proliferation, migration, and invasion of trophoblast cells, therefore, a favorable and unique maternal‐fetal microenvironment is beneficial for fetal survival and development [25, 26]. More importantly, insufficient trophoblast migration and invasion lead to impaired uterine spiral artery reconstruction and are associated with RSA [27]. Meanwhile, several earlier reports investigated cell invasion and migration behavior based on the characteristics of HTR-8/SVneo cell line to evaluate the effects of study subjects on RSA [28-30]. Given that IL-23 was shown to be a key regulator of cell proliferation, migration and invasion, in our study, IL-23 was demonstrated to significantly inhibited the invasion and migration of HTR-8/SVneo cells. In parallel, gelatinases (MMP-2 and MMP-9) play a key role in extracellular matrix remodeling during trophectoderm invasion [31]. In view of this, we also investigated the effects of IL-23 on the expressions of MMP-2 and MMP-9 in trophoblast cells and found that IL-23 decreased the expressions of MMP-2 and MMP-9 in a concentration-dependent manner. These suggest a facilitative effect of high concentrations of IL-23 on the development of RSA.

Additionally, EMT was demonstrated to be extensively involved in a variety of cellular pathophysiological processes, including embryonic development, tissue repair, and cancer metastasis [32, 33]. A growing number of studies have shown that EMT plays an important role in embryo formation. For example, EMT has been shown to regulate embryonic stem cell differentiation, and the induction of EMT enhanced trophoblast cell invasion and migration [34, 35]. Our results here revealed that IL-23 treatment upregulated E-cadherin expression but downregulated N-cadherin expression, implying the inhibitory effects of IL-23 treatment on EMT in trophoblast cells.

The p38 MAP kinase pathway, which is similar to other MAP kinase cascades, is closely related to inflammation, cell growth, cell differentiation and cell death [36]. It was evidenced that p38 MAPK signaling pathway can be activated by pro-inflammatory cytokines such as interleukins and TNF-α [15]. Studies have found that in RSA, when MAPK/p38 signaling pathway was activated, trophoblast cell invasion was attenuated [37, 38]. In the present study, we discovered that IL-23 treatment significantly upregulated the expressions of p-p38, p-ERK1/2 and p-JNK, implying that IL-23 activated p38 MAPK signaling pathway, which was consistent with the results we mentioned previously [11]. To investigate IL-23 action on the p38 MAPK signaling pathway, we employed SB239063 on trophoblast cells. SB239063 is a potent inhibitor of p38 MAP kinase, exhibiting specific and high-affinity binding to p38 MAP kinase, resulting in effective inhibition of its activity [39]. Also, in early human pregnancy, investigators have studied the role of NOD1 and NOD2 in controlling trophoblast invasion through the MAPK/p38 signaling pathway by using this inhibitor [37]. In this study, although the functional activity of HTR-8/SVneo cells was significantly reduced by the increase in IL-23, the addition of SB239063 inhibited p38 MAPK activity while leading to improvement of HTR-8/SVneo cell function. Again, it was demonstrated that IL-23 can activate and function in the p38 MAPK signaling pathway.

To sum up, to the best of our knowledge, the present study is the first to demonstrate that IL-23 and its receptor were highly expressed in IL-23-treated trophoblast cells. The finding that IL-23 treatment inhibited proliferation, invasion, migration and EMT of trophoblast HTR-8/SVneo cells via activating p38 MAPK signaling pathway suggests the novelty of the present study and provides a new promising target for therapeutic intervention in RSA.
